# Effect of Imidazole as Corrosion Inhibitor on Carbon Steel Weldment in District Heating Water

**DOI:** 10.3390/ma14164416

**Published:** 2021-08-06

**Authors:** Sang-Jin Ko, Seok-Ryul Choi, Min-Sung Hong, Woo-Cheol Kim, Jung-Gu Kim

**Affiliations:** 1 School of Advanced Materials Science and Engineering, Sungkyunkwan University, 2066, Seobu-ro, Jangan-gu, Suwon 16419, Korea; tkdwls1315@naver.com (S.-J.K.); seokryul08@gmail.com (S.-R.C.); smith803@skku.edu (M.-S.H.); 2 Technical Efficiency Research Team, Korea District Heating Corporation, 92 Gigok-ro, Yongin 06340, Korea; kwc7777@kdhc.co.kr

**Keywords:** carbon steel, welding, corrosion, corrosion inhibitor, imidazole

## Abstract

Many research studies have been conducted on the corrosion inhibition performance of imidazole in acidic environments such as in the piping of a petrochemical plant. However, there has been no study on the effect of imidazole in alkaline conditions such as a local district water heating environment. Therefore, in this study, the effect of imidazole as a corrosion inhibitor on carbon steel weldment was investigated in alkaline district heating water. Inhibition efficiency and electrochemical properties were investigated by potentiodynamic polarization test and electrochemical impedance spectroscopy. As the concentration of imidazole increased up to 500 ppm, inhibition efficiency increased up to 91.7%. At 1000 ppm, inhibition efficiency decreased. Atomic force microscopy showed that surface coverage of imidazole at 1000 ppm is lower than that of imidazole at 500 ppm. X-ray photoelectron spectroscopy showed that with 500 ppm of imidazole, the amount of pyrrole type interaction is 4.8 times larger than pyridine type interaction. At 1000 ppm of imidazole, the amount of pyridine type interaction is 3.49 times larger than pyrrole type interaction. Depending on the concentration of imidazole, the ratio of interaction between carbon steel and imidazole affected inhibition efficiency.

## 1. Introduction

Corrosion of metal in aqueous systems has led to structural degradation and accidents. In huge fluid transport systems such as those used in the petrochemical industry and district heating systems, the effect of corrosion is more extensive because it is hard to use expensive high corrosion-resistant metals in such large systems [1,2,3,4,5]. The corrosion in a district heating system directly affects the lifespan and function of pipes by causing metal ion solvation and corrosion byproducts. Weldments of carbon steel are especially susceptible due to properties such as having different microstructures of base metal [6]. 

To reduce this problem, low-cost water treatment methods have been applied including pH control, deaeration, and addition of inhibitors [7,8,9]. The organic inhibitor is one of the major methods used to reduce corrosion rate by its adsorbing on metal surfaces [10]. Organic inhibitors that have S, N, O, P, and ring structures are known as heterocyclic compounds [10]. However, many types of corrosion inhibitors have low biodegradability and cause environmental problems due to toxic properties [11]. Therefore, research has been actively conducted on environmentally friendly corrosion inhibitors [12,13,14]. Among them, imidazole and its derivatives, considered “green” corrosion inhibitors with nontoxic properties, have been studied as corrosion inhibitors for iron and copper [15,16,17,18,19,20]. Many studies have been conducted on the corrosion inhibition performance of imidazole in acidic environments such as in the piping of a petrochemicals plant [21,22]. However, there is no study on the effect of imidazole in alkaline conditions such as the local district heating environment. 

In this study, we, therefore, analyzed the effect of imidazole on the weldment of carbon steel in deaerated district heating water with electrochemical analysis techniques such as potentiodynamic polarization test and electrochemical impedance spectroscopy to obtain corrosion rate and inhibition efficiency. By using surface analysis such as atomic force microscopy (AFM) and X-ray photoelectron spectroscopy (XPS), we confirmed the adsorption of imidazole on the metal surface and analyzed the form of adsorption. Ultimately, we achieved the optimum concentration of imidazole and analyzed the mechanism of inhibition.

## 2. Materials and Methods

### 2.1. Specimens and Solution Preparation

**Figure 1:**
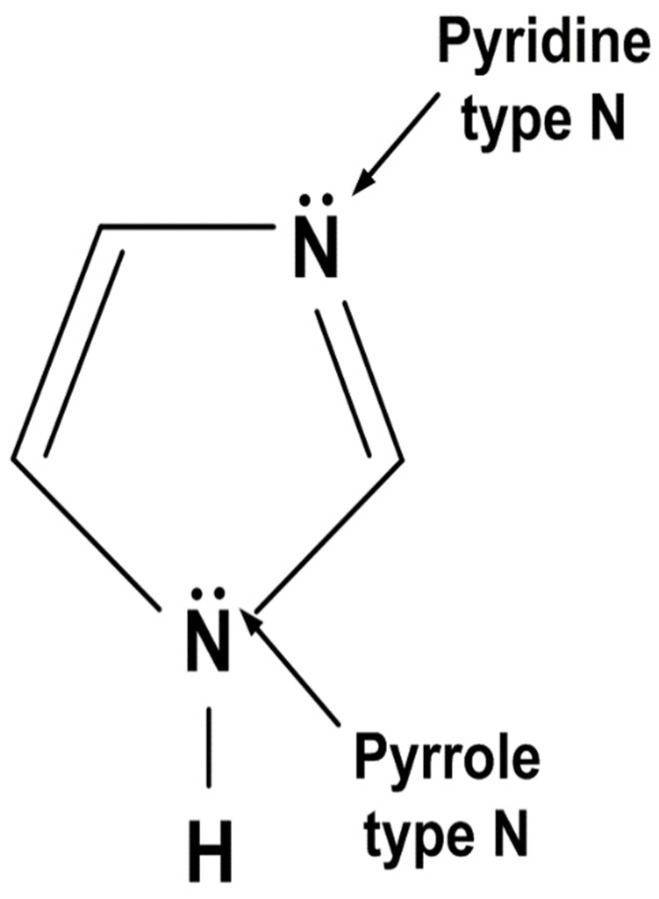
Molecular structure of imidazole.

All the electrochemical measurements depending on imidazole concentration were conducted on carbon steel including the welding bead. SPPS-38 (ASTM A135) was used for carbon steel and chemical composition was as follows: 0.25C-0.35Si-0.3Mn-0.04P-0.04S-Fe (wt.%). A welded joint used for this work was manufactured by multi-pass gas tungsten arc welding (GTAW) with ER70S-G filler. The chemical and physical properties of the welded joint can be changed by the welding operator and circumstances when welding was performed. For this reason, the five weldment specimens used in this work were machined from the same welded joint. Figure 1 shows the molecular structure of imidazole, purchased from Sigma-Aldrich (ST. Louis, MO, USA). The surface was polished with SiC papers starting from 100 grit and progressing to 600 grit size, rinsed with ethanol, and dried with air. After that, the surface of the specimens was covered with silicone rubber except for a 0.24 cm^2^ area (0.8 cm × 0.3 cm). The controlled area included weldment, heat-affected zone, and base metal. The controlled specimen was exposed to deaerated district heating water with 0, 100, 300, 500, and 1000 ppm (mg/L) of imidazole at 60 °C and pH 10 conditions. To make up the solution, HCl, Mg(OH)_2_, CaCO_3_, Fe_3_O_4_, and NH_4_OH were added. The chemical composition of the solution is listed in Table 1. The pH of the solution was controlled by 0.1 M NaOH solution. The surface for the immersion test was polished with SiC papers up to 2000 grit size, rinsed with deionized (DI) water, and dried in the air. Afterward, acid cleaning was conducted with 500 mL of HCl, 500 mL of DI water, and 3.5 g of hexamethylenetetramine.

### 2.2. Electrochemical Measurement

The electrochemical properties of the specimen were evaluated by potentiodynamic (PD) and electrochemical impedance spectroscopy (EIS) tests. All tests were performed using multi-potentiostat/galvanostat model VSP-300 (BioLogic, Seyssinet-Pariset, France) after 6 h immersion in the tested district heating water with imidazole. A conventional three-electrode cell including the prepared carbon steel specimen as the working electrode, two pure graphite counter electrodes, and a saturated calomel electrode (SCE) with a Luggin capillary was used to carry out the PD and EIS tests. With different imidazole concentrations, PD tests were conducted in potential range from −250 mV vs. open-circuit potential (OCP) to 1600 mV vs. SCE at 0.166 mV s^−1^ scan rate. EIS tests were performed under OCP with a sinusoidal amplitude of 20 mV in the frequency range from 100 kHz to 1 mHz. An equivalent circuit was applied to the results of EIS tests and results were analyzed using a proper fitting process by ZsimpWin ver. 3.21 Software (AMETEK scientific instruments, Berwyn, PA, USA).

### 2.3. Surface Analysis 

To identify surface morphology after immersion for 40 h in test solutions, optical microscopy (OM) was conducted for each specimen using LEICA 300 (Leica Microsystems, Langen, Germany). To investigate the bonding type of adsorbed imidazole molecule on the surface of specimens, X-ray photoelectron spectroscopy (XPS) was conducted. XPS was conducted using a SIGMA PROBE (ThermoFisher Scientific, Waltham, MA, USA) in a UHB chamber, equipped with a monochromated Al Kα X-ray source. Specimens were immersed in district heating water conditions containing 500 ppm and 1000 ppm imidazole for 8 h. 

Atomic force microscopy and Kelvin probe force microscopy (KPFM), a mode of AFM, were conducted to identify adsorbed imidazole on a steel surface with topography and surface potential mapping. AFM measurements were conducted with a commercial AFM system (NX-10, Park systems, Suwon, Korea). KPFM measurements were conducted with an AC modulation voltage of 2, the root-mean-square voltage at 17 kHz in the lift mode, and a distance between the tip and sample of 20 nm using a conductive Pt/Cr coated tip (Multi75E-G, BudgetSensors, Sofia, Bulgaria). All measurements were conducted at a 10 μm × 10 μm scale.

## 3. Results and Discussion

### 3.1. Electrochemical Measurement

#### 3.1.1. Potentiodynamic Polarization Tests

**Figure 2:**
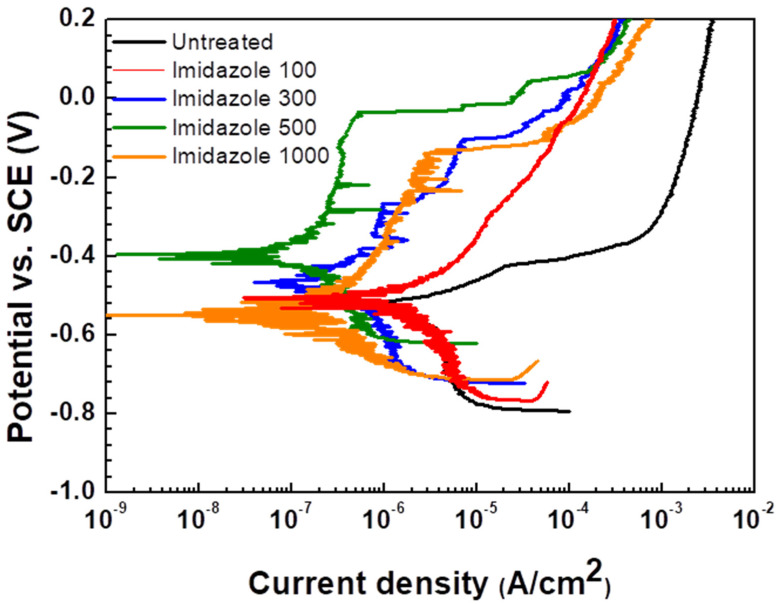
Potentiodynamic polarization curves of SPPS-38 carbon steel weldment as a function of imidazole concentration in the deaerated district heating water at 60 °C.

PD tests were conducted to figure out electrochemical behavior and inhibition efficiency when imidazole is added to district heating water. Figure 2 shows the PD curves of SPPS-38 carbon steel in deaerated district heating water according to the concentration of imidazole at 60 °C. Table 2 presents the results of PD tests. β_c_ is the Tafel slope of cathodic reaction, which is related to cathodic reaction. E_corr_, corrosion potential, is the equilibrium potential where corrosion reaction occurs. It means that corrosion potential is the potential where anodic reaction rate and cathodic reaction rate are equal. I_corr_, corrosion current density, is the current density where anodic reaction rate and cathodic reaction rate are equal. E_b_, breakdown potential, represents the potential where the breakdown of passivity occurs. It means that above E_b_, the current density increases dramatically owing to the propagation of pitting.

The untreated case showed active behavior, while 100 ppm and 300 ppm of imidazole specimens showed active behavior and unstable passive behavior [23], respectively, implying that 100 and 300 ppm of imidazole are not enough to inhibit corrosion effectively. The 500 and 1000 ppm imidazole cases show passive behavior, which means that corrosion on metal surfaces was suppressed by adsorbed imidazole [20,24]. Compared with the untreated case, corrosion current density (i_corr_), which is proportional to corrosion rate, decreased, and corrosion potential (E_corr_) increased with the addition of imidazole. The cathodic current density and cathodic Tafel slope (β_c_) related to cathodic reaction [25] also decreased. These tendencies indicate that imidazole affects both the anodic reaction and the cathodic reaction. Azole-type compounds such as imidazole are known to inhibit the adsorption of hydrogen onto metal surfaces [26]. Furthermore, organic inhibitors that include S, O, and N, such as imidazole, adsorbed on the metallic surface block the active corrosion sites [10]. In this context, increased E_corr_ can be explained in that the degree of decreasing anodic reaction is more than that of decreasing cathodic reaction according to the mixed potential theory [27]. As a result of decreasing anodic and cathodic reactions, the i_corr_ decreases because i_corr_ is where total anodic and cathodic reaction rates are the same. Furthermore, as the concentration of imidazole increases, i_corr_ decreases more according to the characteristics of azole type inhibitors. Azole-type corrosion inhibitors generally show higher inhibition efficiency as the concentration of inhibitor is increased [28,29]. However, this tendency does not fit with the addition of 1000 ppm of imidazole. When 1000 ppm imidazole was added, i_corr_ was higher than 500 ppm imidazole, and the E_corr_ decreased. A different mechanism, therefore, affected the 1000 ppm imidazole case. The breakdown potential (E_b_) represents the potential when breakdown of passivity occurs [30]. The value of E_b_ increases as the concentration of imidazole increases from 300 to 500 ppm but decreases when the concentration of imidazole reaches 1000 ppm. This means that passivity due to imidazole adsorption becomes more stable up to 500 ppm of imidazole, but the passivity of imidazole decreases when 1000 ppm of imidazole was added. It implies that the integrity of adsorbed layer decreases when 1000 ppm of imidazole was added and it can be related to the behavior of imidazole adsorption such as coverage. Meanwhile, inhibition efficiency (IE) was calculated using the following equation [31].

(1) $$\mathrm{IE}\;\left(\%\right)=\frac{i_{uninhibited}-i_{inhibited}}{i_{uninhibited}}\times 100$$

where *i_uninhibited_* is the current density for the case where imidazole is not added, and *i_inhibited_* is the current density for the case where imidazole is added. As expected, 100 ppm and 300 ppm of imidazole show low, at 68.5% and 84.2%, respectively, owing to low adsorption coverage. Moreover, 500 ppm of imidazole shows 91.7% efficiency, the highest value in this experiment, while 1000 ppm of imidazole shows 88.0% of IE, which is slightly lower than the efficiency of 500 ppm, although the concentration of imidazole increased. This implies that the adsorption coverage of imidazole was decreased in 1000 ppm of imidazole solution.

#### 3.1.2. Electrochemical Impedance Spectroscopy

**Figure 3:**
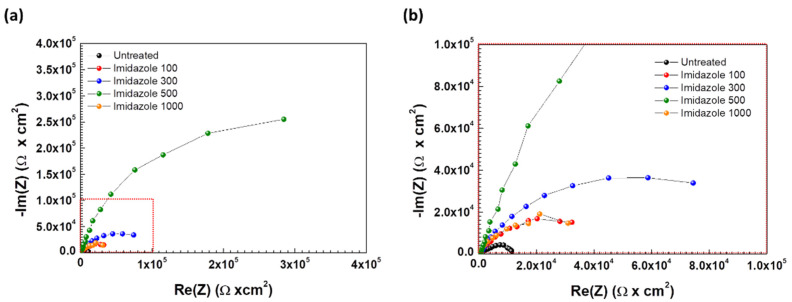
(**a**) Nyquist plots for SPPS-38 carbon steel weldment as a function of imidazole concentration in the deaerated district heating water at 60 °C and (**b**) enlarged image of red dotted square area.

**Figure 4:**
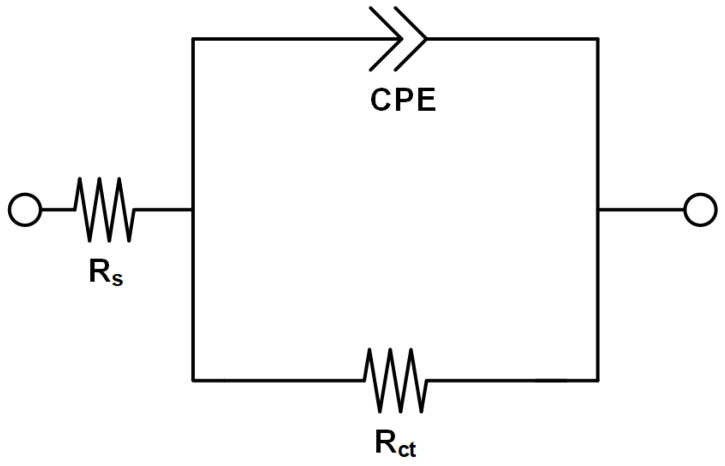
Electrical equivalent circuit for EIS tests.

EIS was conducted to understand electrochemical behavior on metal surfaces depending on imidazole concentration. Figure 3 shows the Nyquist plots obtained for SPPS-38 carbon steel in deaerated district heating water according to the concentration of imidazole at 60 °C. Figure 3 is based on the proper electrical equivalent circuit shown in Figure 4, and the data from EIS tests are listed in Table 3. R_s_ represents the solution resistance, C_dl_ is the capacitance of the electrical double layer, and R_ct_ is the charge transfer resistance. Constant phase element (CPE) is used to express the depression of the Nyquist plot and the parameter including C_dl_ and n_dl_ (0 ≤ n_dl_ ≤ 1), which are the admittance and exponent of CPE, respectively [32]. Depression phenomenon, the deviation from the ideal capacitive behavior, is expressed by n_dl_ in this circuit. When the value of n_dl_ is close to 1, it implies that the property of the capacitor becomes ideal.

**Figure 5:**
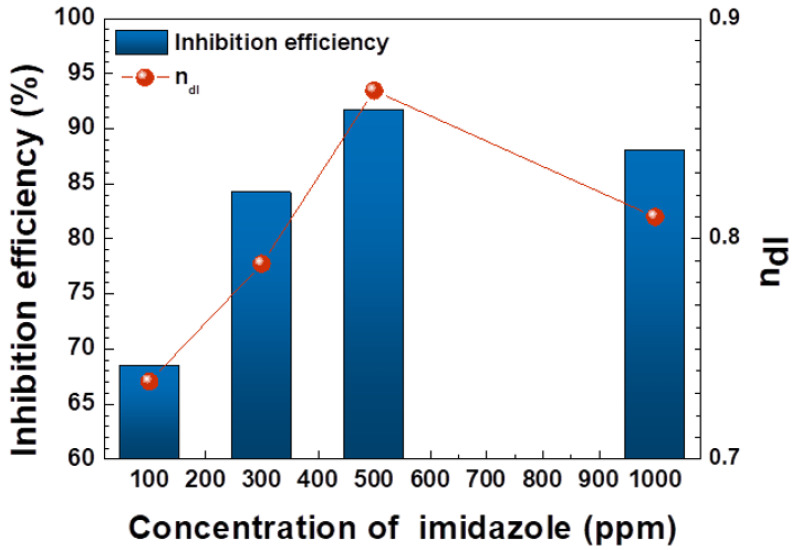
Inhibition efficiency (%) from potentiodynamic polarization test and value of n_dl_ from EIS.

In the case of 500 ppm imidazole, the C_dl_ value is the lowest, and the n, R_ct_ values are the highest. The values of C_dl_ can be expressed as C = εA/t where ε is the permittivity of the material, A is the area of the conductive plate, and d is the thickness of filled material, which has ε. The lowest value of C_dl_ means that the average thickness of the electrical double layer is increased due to the adsorbed imidazole. In other words, the distance between the metal surface and the outer Helmholtz plane is increased. Increasing n_dl_ means that the coverage of imidazole is increased. As a result, the value of R_p_, polarization resistance, or R_ct_ in this circuit is the highest at 500 ppm of imidazole. Furthermore, inhibition efficiency was calculated from EIS data with the following equation [33].

(2) $$\mathrm{IE}\left(\%\right)=\frac{\frac{1}{R_{pu}}-\frac{1}{R_{pi}}}{\frac{1}{R_{pu}}}\times 100\;$$

where *R_pu_* is the *R_p_* of the uninhibited case, and *R_pi_* is the *R_p_* when the specimen was inhibited by imidazole. In Figure 5, the IE calculated with EIS data show similar values to the IE calculated by PD tests. In other words, the value of IE is the highest at 500 ppm of imidazole. The value of n_dl_, which means the integrity of capacitor, is the highest at 500 ppm of imidazole. Thus, the coverage of imidazole on the metal surface and integrity of capacitor affected by the adsorption of imidazole are the highest in 500 ppm of imidazole. However, the results of EIS for 1000 ppm of imidazole trend are negative, compared to results at 500 ppm. The value of C_dl_ increased and R_ct_, n_dl_, and IE decreased, which means, respectively, that coverage of imidazole on the metal surface and resistance to corrosion were decreased. These tendencies are consistent with the results of PD tests.

### 3.2. Surface Analysis

#### 3.2.1. Optical Microscopy (OM)

**Figure 6:**
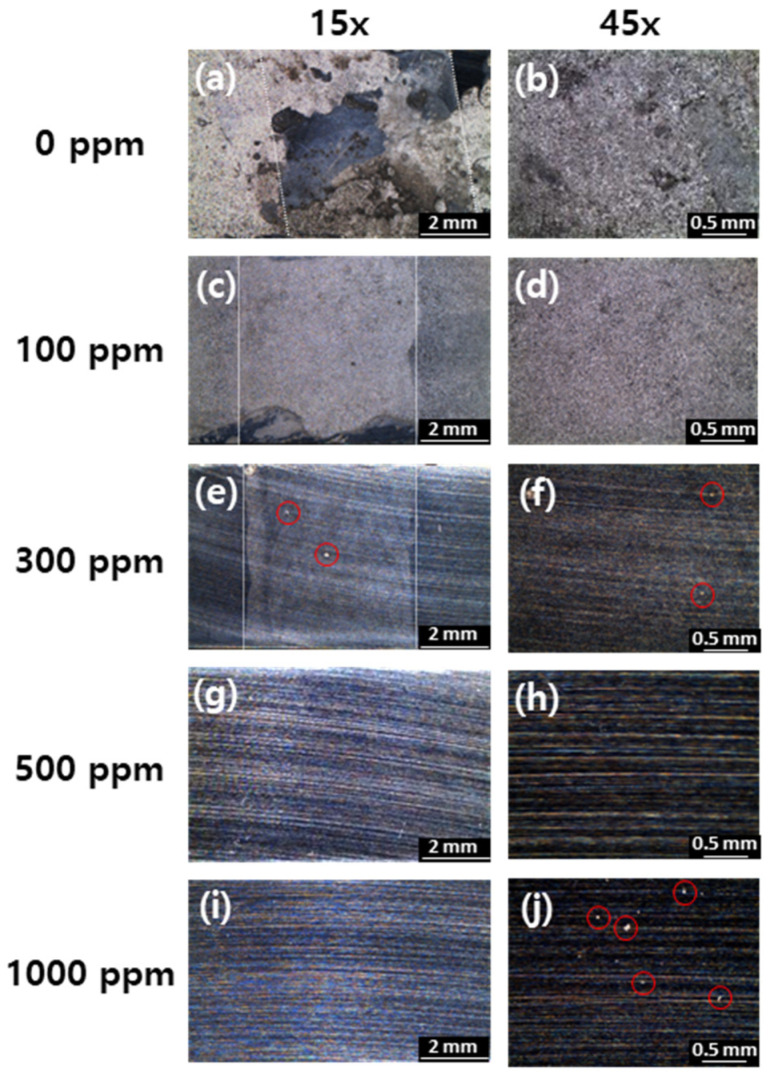
Optical microscopy images (15×) of carbon steel weldment after 40 h immersion in the solution with addition of (**a**) 0 ppm, (**c**) 100 ppm, (**e**) 300 ppm, (**g**) 500 ppm, and (**i**) 1000 ppm of imidazole and magnified images (45×) of (**b**) 0 ppm, (**d**) 100 ppm, (**f**) 300 ppm, (**h**) 500 ppm, and (**j**) 1000 ppm of imidazole; white dotted lines show weldment area and red circles show micro-scale pitting.

To clarify the effect of imidazole as a corrosion inhibitor and to observe surface morphology after corrosion, surface images were obtained using OM after 40 h immersion in the solutions of 0, 100, 300, 500, and 1000 ppm of imidazole. When imidazole was not added, the surface corroded more uniformly and severely than in the other cases, as shown in Figure 6a, and the welded area was distinct owing to its different microstructure and electrochemical behavior, compared to base metal [34]. When 100 ppm of imidazole was added, uniform corrosion also occurred and the welded area become clearly visible. The adsorption and the effect of inhibition were insufficient to protect from corrosion. In the case of 300 ppm, degradation of the surface was notably decreased, but the welded area can still be distinct and micro-scale pitting was observed in the red circles in Figure 6e,f. However, when either 500 or 1000 ppm of imidazole was added, the metals barely corroded even the polishing lines could be visible. It means that the weldment areas were not visible due to higher corrosion resistance as shown in Figure 6g,i. However, in the case of 1000 ppm, micro-scale pittings were observed in red circles of Figure 6j. These results are consistent with electrochemical measurements. When 1000 ppm of imidazole was added, the coverage of imidazole on the metal surface, and resistance to corrosion was decreased, compared to 500 ppm of imidazole.

#### 3.2.2. X-ray Photoelectron Spectroscopy (XPS)

**Figure 7:**
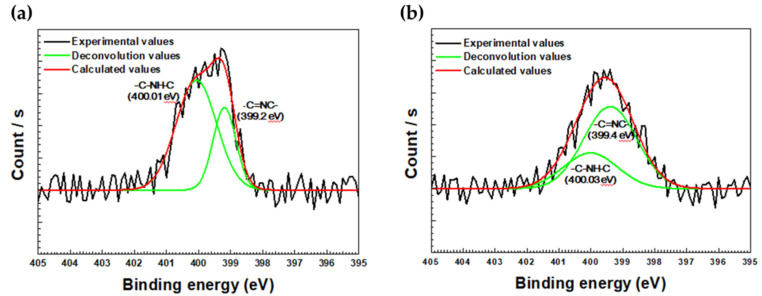
XPS results (N1s) of carbon steel weldment after 6 h immersion in (**a**) 500 ppm and (**b**) 1000 ppm of imidazole.

XPS was conducted to investigate how imidazole adsorbed onto the carbon steel surface. Figure 7 and Table 4 are deconvoluted results of XPS and the area ratios between deconvoluted -C=NC (pyridine type N, 399.44 eV) and -C-NH-C (pyrrole type N, 400.1 eV) peaks from N1s XPS results [35]. In Figure 7, -C=NC and -C-NH-C peaks, which can be found in imidazole, are shown in both 500 and 1000 ppm of imidazole. Imidazole is therefore adsorbed on metal surfaces in the form of -C=NC and -C-NH-C interaction. In the case of 500 ppm, the area under the -C-NH-C peak is 4.8 times greater than the area under the -C=NC peak. However, in the case of 1000 ppm, the area under -C=NC is 3.49 times greater than that under the -C-NH-C peak. In the 500 ppm imidazole sample, -C-NH-C pyrrole type N is therefore predominant. In the 1000 ppm imidazole sample, -C=NC pyridine type N is predominant.

#### 3.2.3. Atomic Force Microscopy (AFM)

**Figure 8:**
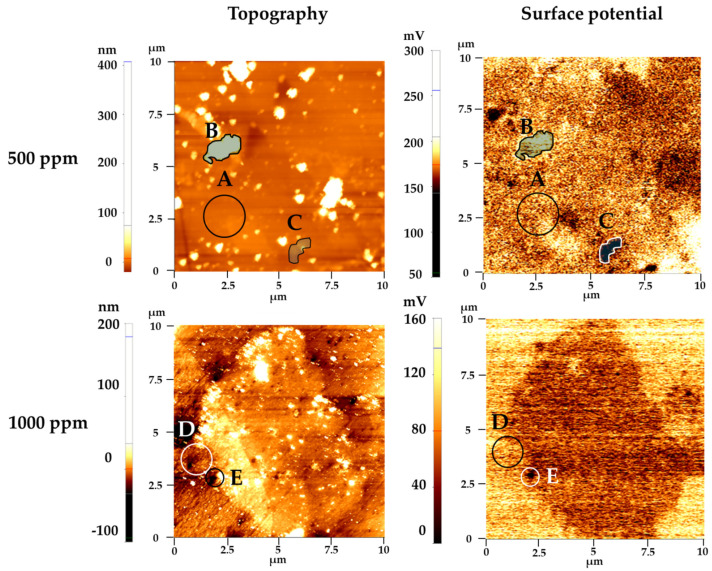
Topography and surface potential of carbon steel weldment after 6 h immersion in 500 and 1000 ppm of imidazole solution as measured by AFM with SKPFM mode.

AFM study was conducted to investigate surface properties of carbon steel weldment after 6 h immersion in 500 and 1000 ppm of imidazole. Results are shown in Figure 8. In this analysis, the difference of surface potential in each measurement was used because the value of surface potential can change depending on the condition of the AFM tip in each measurement. In Figure 8, we delineate several areas in the 500 and 1000 ppm cases. In 500 ppm, three distinct areas were observed depending on surface potential and are indicated by A, B, and C. The measured surface potential of each area came out as B > A > C and each difference is listed in Table 5. The A site occupies the greatest surface area and shows relatively medium surface potential and topography. The B site showed protruded topography and the highest surface potential among the three locations. The C site showed the lowest surface potential. The surface potential measured by KPFM can be correlated with corrosion potential and corrosion behavior [36,37,38]. Site C, therefore, acted as an anodic site where oxidation reaction occurred. According to the topography and surface potential, site A is the area where the metal surface was protected by adsorbed imidazole. Site B shows iron oxide with particle shape, and site C is the area with an unprotected metal surface. At 1000 ppm, two distinct areas were observed depending on surface potential and are indicated by D and E. Surface potential of D is 36 mV larger than E, similar to the difference in potential between A and C. It implies that D is also an area protected by imidazole and that E is an unprotected area, which served as an anodic site for corrosion. The reason why protrusion, which is estimated as iron oxide in 1000 ppm, does not show the higher surface potential is that too little iron oxide is present to affect surface potential considerably. Compared to the 500 ppm sample, the unprotected surface area that acts as the anodic site for corrosion is more expansive in the 1000 ppm sample. The surface coverage of imidazole on carbon steel is, therefore, lower in 1000 ppm than in 500 ppm, which is consistent with the results of electrochemical measurement and XPS.

### 3.3. Inhibition Mechanism

**Figure 9:**
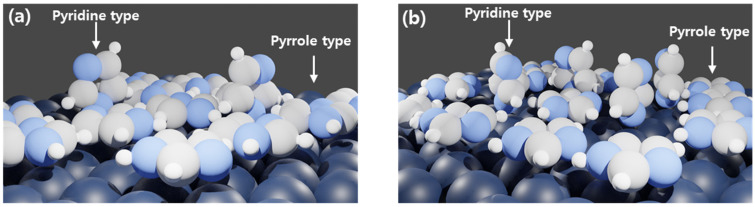
Schematic view of imidazole adsorption at different concentrations of imidazole: (**a**) 500 ppm; (**b**) 1000 ppm.

In general, the IE of azole-type corrosion inhibitors increases as the concentration of inhibitor increases [28,29]. In this case, however, there is a concentration of imidazole that provides maximum IE, and beyond that concentration, the IE decreased. Due to higher surface coverage, pyrrole type interaction in which imidazole adsorbs to the metal surface lying down has been known as the more efficient corrosion inhibitor than pyridine type interaction in which imidazole adsorbs standing up [35]. Up through 500 ppm concentration of imidazole, both interactions decrease corrosion rate by covering and shielding the metal surface. However, when imidazole concentration is increased to 1000 ppm, the trend is changed. When pH of solution is more than 7, ImiH(aq) = Imi^−^(aq) + H^+^(aq) becomes the dominant reaction and deprotonated imidazole (Imi^−^(aq)) is formed [32]. Then, neutral form of imidazole ImiH(aq) and Imi^−^(aq) coexist in the solution with a ratio of 10^4.52^: 1 [39]. Pyrrole type interaction is stable when ImiH(aq) is adsorbed on the Fe matrix, with −0.83 eV of adsorption energy. Pyridine interaction is more stable when Imi^−^(aq) is adsorbed on the Fe matrix with −1.96 eV of dissociative chemisorption energy [33]. Furthermore, Imi^−^(aq) has a greater pro bability for adsorption than ImiH(aq) due to its low energy for adsorption [40]. For this reason, as the concentration of imidazole increased to 1000 ppm, the probability of Imi^−^(aq) adsorption increased, and the proportion of pyridine type interaction increased. As a result, the coverage and inhibition efficiency slightly decreased according to adsorption type, as shown in Figure 9.

## 4. Conclusions

In this paper, the performance of imidazole as a corrosion inhibitor in district heating water was evaluated, and the mechanism of changing efficiency depending on the concentration of imidazole was investigated.

- In electrochemical measurement, the inhibition efficiency of imidazole on weldment of carbon steel increased as the concentration of imidazole increased up to peak inhibition efficiency at 500 ppm in this experiment;
- Inhibition efficiency decreased from peak value when imidazole concentration reached 1000 ppm;
- OM measurement after 40 h immersion showed that 500 ppm imidazole solution offered the highest inhibition efficiency and corrosion resistance. In 300 and 1000 ppm solution samples, micro-scale pittings were observed implying the lower inhibition efficiency and corrosion resistance;
- XPS analysis showed that the pyrrole type interaction between imidazole and metal surface was more abundant in 500 ppm solution and pyridine type interaction was more abundant in 1000 ppm solution. Pyrrole-type interaction is known to offer higher inhibition efficiency due to higher coverage;
- AFM measurement also showed that surface coverage of imidazole is lower in 1000 ppm samples;
- Electrochemical measurement, immersion tests, and AFM showed the lower inhibition efficiency in the solution with 1000 ppm of imidazole. XPS and prior studies showed that as the concentration of imidazole increased, pyridine interaction between imidazole and Fe substrate also increased, which result in the decrease of coverage and inhibition efficiency.

Therefore, samples from 1000 ppm of imidazole solution show a lower inhibition efficiency than samples from 500 ppm of imidazole.

## Figures and Tables

**Table 1:** Chemical composition of the tested district heating water (mg∙L^−1^).

pH	Cl^−^	Mg^2+^	Ca^2+^	Fe^2+^, Fe^3+^	NH_4_^+^
10.0	14.6	0.2	1.06	0.2	1.53

**Table 2:** Parameters extracted from the potentiodynamic polarization curves of SPPS-38 carbon steel weldment in the deaerated district heating water containing different concentrations of imidazole at 60 °C.

Imidazole Conc. (ppm)	−β_c_ (mV/decade)	E_corr_ (mV_SCE_)	i_corr_ (μA/cm^2^)	E_b_ (mV_SCE_)	IE (%)
0	698.8	−519.875	2.908		-
100	357.4	−506.271	0.916		68.5
300	322.5	−466.211	0.46	−268.0	84.2
500	280.4	−402.451	0.24	−33.6	91.7
1000	135.1	−551.359	0.35	−130.0	88.0

**Table 3:** Parameters extracted from the EIS data of SPPS-38 carbon steel weldment in the deaerated district heating water containing different concentrations of imidazole at 60 °C.

Imidazole Conc. (ppm)	R_s_ (kΩ∙cm^2^)	C_dl_ (μF/cm^2^)	n_dl_	R_ct_ (Ω∙cm^2^)	R_p_ (Ω∙cm^2^)	IE (%)
0	264.25	2.875 × 10^−5^	0.6105	1.38 × 10^4^	1.38 × 10^4^	-
100	263.25	3.285 × 10^−5^	0.7353	4.89 × 10^4^	4.89 × 10^4^	71.8
300	713.25	1.237 × 10^−5^	0.7888	1.04 × 10^5^	1.04 × 10^5^	86.8
500	600.25	5.130 × 10^−6^	0.8673	6.02 × 10^5^	6.02 × 10^5^	97.7
1000	189.12	5.642 × 10^−5^	0.8747	4.01 × 10^4^	4.01 × 10^4^	89.7

**Table 4:** The area ratio between –C=NC and –C-NH-C peaks from N_1s_ XPS results.

	500 ppm	1000 ppm
-C=NC (399.44 eV)	1	3.49
-C-NH-C (400.1 eV)	4.8	1

**Table 5:** Difference of surface potential between distinctive areas in results of AFM measurement.

Imidazole Conc. of Solution	Location	Surface Potential Difference(mV)
500 ppm	A-B	−23
	B-C	63
	A-C	38
1000 ppm	D-E	39

## Data Availability

Data sharing is not applicable for this article.
